# Novel Carbazole Skeleton-Based Photoinitiators for LED Polymerization and LED Projector 3D Printing

**DOI:** 10.3390/molecules22122143

**Published:** 2017-12-04

**Authors:** Assi Al Mousawi, Patxi Garra, Frédéric Dumur, Thanh-Tuan Bui, Fabrice Goubard, Joumana Toufaily, Tayssir Hamieh, Bernadette Graff, Didier Gigmes, Jean Pierre Fouassier, Jacques Lalevée

**Affiliations:** 1 Institute of Science and Materials of Mulhouse IS2M—UMR, The National Center for Scientific Research (CNRS) 7361—UHA, 15, Rue Jean Starcky, 68057 Mulhouse CEDEX, France; assimoussawi@gmail.com (A.A.M.); patxi.garra@uha.fr (P.G.); bernadette.graff@uha.fr (B.G.); jean-pierre.fouassier@uha.fr (J.P.F.); 2 Laboratory of Materials, Catalysts, Environment and Analytical Methods (MCEMA-CHAMSI), EDST, Lebanese University, Campus Hariri, Hadath, Beyrouth, Lebanon; joumana.toufaily@ul.edu.lb (J.T.); tayssir.hamieh@ul.edu.lb (T.H.); 3 Institut de Chimie Radicalaire—CNRS, Aix-Marseille University, UMR 7273, F-13397 Marseille, France; dider.gigmes@univ-amu.fr; 4 Laboratory of Physico-chemistry of Polymers and Interfaces LPPI, University of Cergy-Pontoise, 5 Mail Gay Lussac, Neuville-sur-Oise, 95031 Cergy-Pontoise CEDEX, France; tbui@u-cergy.fr (T.-T.B.); fabrice.goubard@u-cergy.fr (F.G.)

**Keywords:** cationic polymerization, free radical polymerization, light emitting diodes (LEDs), photoinitiators, 3D printing

## Abstract

Radical chemistry is a very convenient way to produce polymer materials. Here, an application of a particular photoinduced radical chemistry is illustrated. Seven new carbazole derivatives **Cd1**–**Cd7** are incorporated and proposed as high performance near-UV photoinitiators for both the free radical polymerization (FRP) of (meth)acrylates and the cationic polymerization (CP) of epoxides utilizing Light Emitting Diodes LEDs @405 nm. Excellent polymerization-initiating abilities are found and high final reactive function conversions are obtained. Interestingly, these new derivatives display much better near-UV polymerization-initiating abilities compared to a reference UV absorbing carbazole (CARET 9*H*-carbazole-9-ethanol) demonstrating that the new substituents have good ability to red shift the absorption of the proposed photoinitiators. All the more strikingly, in combination with iodonium salt, **Cd1**–**Cd7** are likewise preferred as cationic photoinitiators over the notable photoinitiator bis(2,4,6-trimethylbenzoyl)phenylphosphine oxide (BAPO) for mild irradiation conditions featuring their remarkable reactivity. In particular their utilization in the preparation of new cationic resins for LED projector 3D printing is envisioned. A full picture of the included photochemical mechanisms is given.

## 1. Introduction

Radical (photo) chemistry plays a significant role in the production of polymer materials. Due to their advantages, compared to thermal activation and their applications as a green technology, light activated polymerization reactions are experiencing an ever increasing development and have inspired a lot of work on the design of photoinitiators PIs and multicomponent photoinitiating systems PISs (see e.g., in recent papers and books [1,2,3,4,5,6,7,8,9,10,11,12,13,14] and references therein). One current approach concerns the adaptation of PIs to LED irradiation wavelengths and, among others, a particularly interesting application refers to 3D printing. PIs are able to produce radicals or cations under light exposure. Suitable PISs can have dual behavior: using the same system both a radical and a cationic photopolymerization can be carried out separately and/or together (like interpenetrating polymer networks or IPNs).

**Scheme 1:**
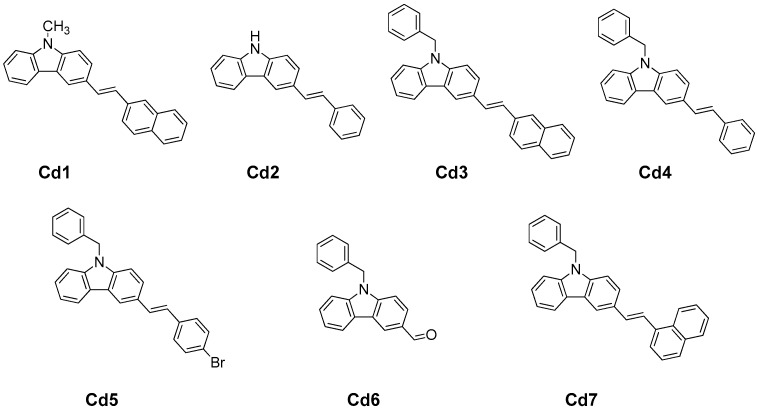
The different carbazole derivatives (Cds) (denoted **Cd1**–**Cd7**) investigated in this study.

In a recent study [15], four new carbazole derivatives exhibiting long wavelength absorption have been proposed as PIs. As this kind of skeleton was particularly promising, seven new carbazole derivatives (Cds) **Cd1**–**Cd7** exhibiting long wavelength absorption thanks to well selected substituents were synthesized (see Scheme 1); 9*H*-carbazole-9-ethanol (CARET) used previously in our papers [16,17,18] was chosen here as a reference carbazole derivative. The new carbazole derivatives were incorporated into two-component (PI/iodonium salt Iod) and sometimes into three-component (PI/Iod/amine) photoinitiating systems (PISs) to induce the formation of reactive species (radicals or radical cations) for free radical polymerization (FRP) and/or cationic polymerization (CP) under near-UV and visible light. Interestingly, the different substituents introduced on the carbazole skeleton highly affect the absorption as well as the photochemical/electrochemical properties of **Cd1**–**Cd7**. Obviously, this will pave the way to study the structure/reactivity/efficiency relationships of the studied carbazole derivatives as photoinitiators in CP and FRP. The use of these new high performance photosensitive systems in LED projector 3D printing experiments is also described. Finally, an insight into the involved mechanisms is provided.

## 2. Results and Discussion

### 2.1. Experimental Results

#### 2.1.1. Light Absorption Properties of **Cd1**–**Cd7**

**Figure 1:**
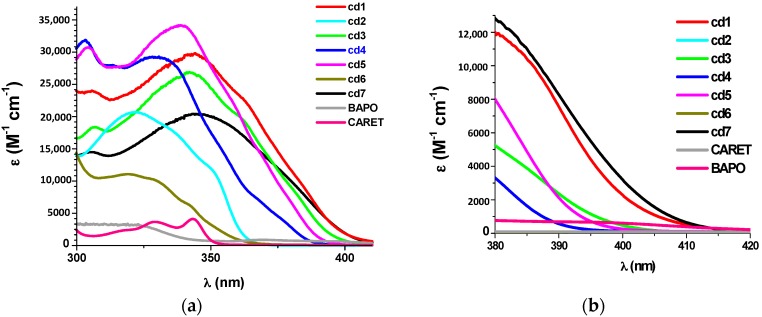
Absorption properties for **Cd1**–**Cd7** vs. CARET and BAPO. (**a**) For short wavelength (300–400 nm); (**b**) for extended wavelengths, particularly between 380 and 415 nm.

**Figure 2:**
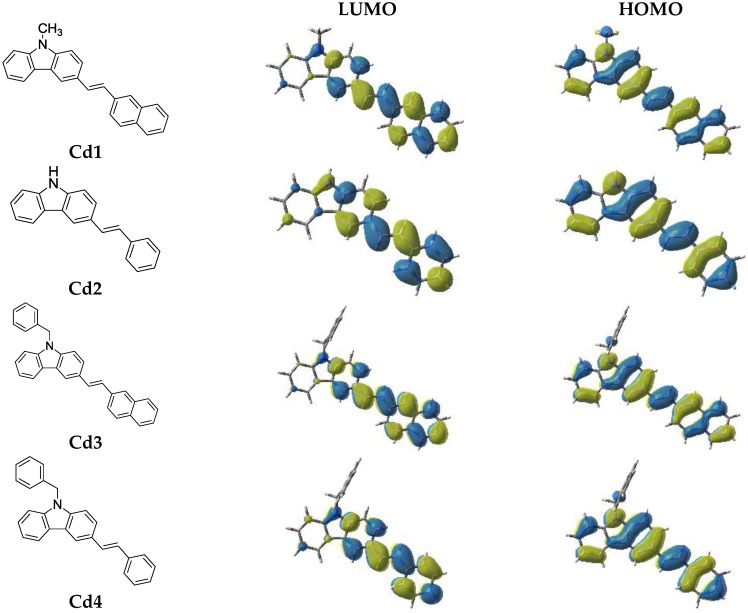

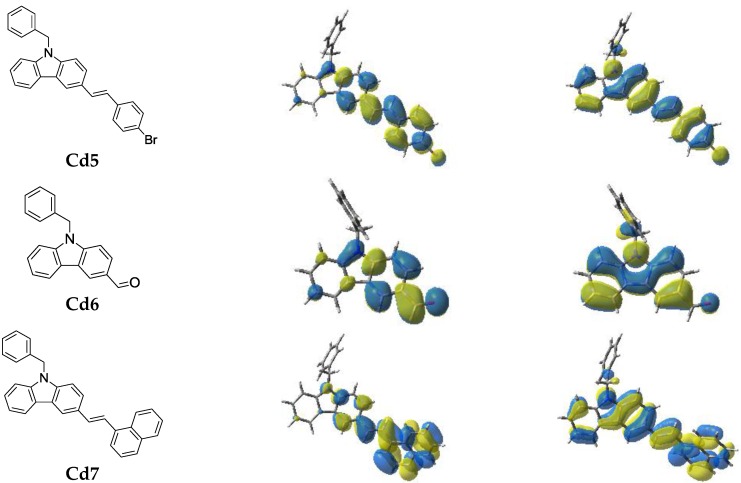
Contour plots of HOMOs and LUMOs for the Cd structures optimized at the B3LYP/6-31G* level of theory.

The ground state absorption spectra of the new proposed photoinitiators in dichloromethane (DCM) are shown in Figure 1 (see additionally Table 1). These compounds exhibit high molar extinction coefficients in the near UV (e.g., **Cd7** ~20,500 M^−1^·cm^−1^ @345 nm and 1500 M^−1^·cm^−1^ @405 nm). Their absorptions in the 350–410 nm spectral range are interesting, guaranteeing a good overlap with the emission spectrum of the LED@405 nm used in this work. The optimized geometries and the frontier orbitals (Highest Occupied Molecular Orbital—HOMO and Lowest Unoccupied Molecular Orbital—LUMO) are shown in Figure 2. Both the HOMO and LUMO are firmly delocalized all over the π system clearly showing a π→π* lowest energy transition. The extension of the π framework by means of various substituents on the carbazole moiety causes a reasonable shift in the spectrum (e.g., the presence of 3-(1-naphthylethenyl) group causes a bathochromic shift in the spectrum of **Cd7**, Figure 1, and Table 1). A significant difference in the HOMO structure of **Cd3** and **Cd7** is also observed. The clear bathochromic shift caused by the different substituents in **Cd1**–**Cd7** is consummately anticipated and it follows the following order: **Cd7**~**Cd1** > **Cd3** > **Cd5** > **Cd4** > **Cd2** > **Cd6**. For the reference carbazole derivative (CARET), with no substituent on the phenyl rings, a shorter wavelength absorption is found as well as lower extinction coefficients (e.g., no absorption for CARET@405 nm vs. ε ~1500 (M^−1^·cm^−1^) for **Cd7** see in Table 1). Surprisingly, contrasted with the well known photoinitiator (BAPO), the new proposed structures **Cd1**–**Cd7** exhibit much better light absorption properties in all the 350–410 nm range (Figure 1).

#### 2.1.2. Cationic Photopolymerization (CP) of Epoxides

**Figure 3:**
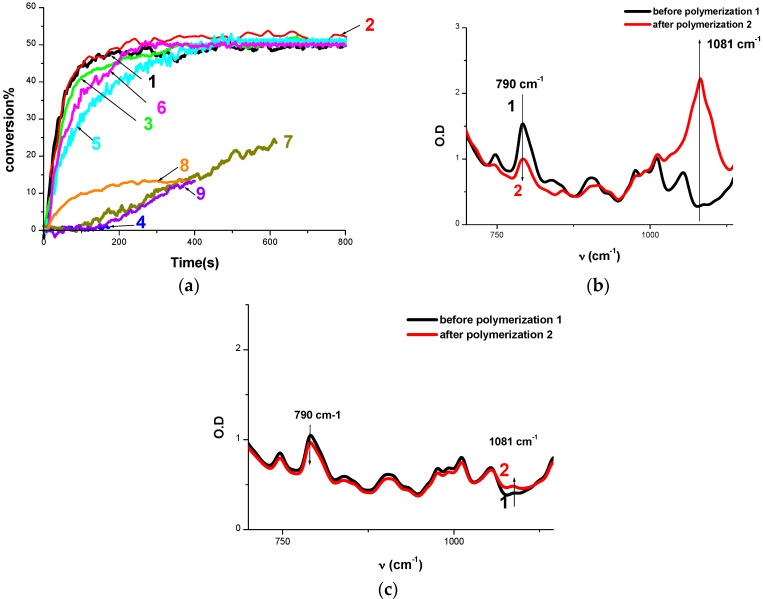
(**a**) Polymerization profiles of EPOX (epoxy function conversion vs. irradiation time) upon exposure to the LED@405 nm under air in the presence of different photoinitiating systems: (1) **Cd7**/Iod (0.5%/1% *w*/*w*); (2) **Cd1**/Iod (0.5%/1% *w*/*w*); (3) **Cd3**/Iod (0.5%/1% *w*/*w*); (4) **Cd2**/Iod (0.5%/1% *w*/*w*); (5) **Cd4**/Iod (0.5%/1% *w*/*w*); (6) **Cd5**/Iod (0.5%/1% *w*/*w*); (7) **Cd6**/Iod (0.5%/1% *w*/*w*); (8) BAPO/Iod (0.5%/1% *w*/*w*); (9) CARET/Iod (1%/1% *w*/*w*); IR spectra recorded before and after polymerization using: (**b**) **Cd7**/Iod (0.5%/1% *w*/*w*); (**c**) CARET/Iod (1%/1% *w*/*w*). The irradiation starts at *t* = 10 s.

Upon LED@405 nm illumination, the cationic polymerization (CP) of epoxides (e.g., EPOX) under air using two-component photoinitiating systems based on Cd/Iod blends (0.5%/1% *w*/*w*) displays high effectiveness in term of final epoxy function conversion (FC) (e.g., FC 55% with Cd7; at *t* = 800 s; Figure 3a, band 1). The polyether network formation can clearly be observed at ~1080 cm^−1^ (Figure 3b) in the FTIR spectra. Iod alone does not initiate the polymerization, obviously demonstrating the role of **Cd1**–**Cd7** in inducing the iodonium salt disintegration upon near UV light LED. Amazingly, high rates of polymerization (Rp) were obviously achieved with the **Cd1**–**Cd7**/Iod systems aside from **Cd2**/Iod and **Cd6**/Iod system contrasted with the CARET/Iod system set as a reference for which no polymerization happens. The correlation of **Cd1**–**Cd7** with CARET is important as all photoinitiators are characterized by having of a carbazole moiety. Likewise, astoundingly, in comparison with the well-known BAPO/Iod (0.5%/1% *w*/*w*) system, both **Cd1** and **Cd7**, aside from **Cd2** and **Cd6**, are discovered much preferable photoinitiators over BAPO (Figure 3a, curve 8 for BAPO contrasted with curve 1 for **Cd7**). This information demonstrates the outstanding predominance of the new carbazoles as far as efficiencies over the well-known BAPO (i.e., the polyether peaks are not observed neither for CARET/Iod e.g., in Figure 3c, nor for BAPO/Iod contrasted with the **Cd7**/Iod system as e.g., in Figure 3b). The efficiency trend for CP using LED@405 nm follows the following order: **Cd7**~**Cd1** > **Cd3** > **Cd5** > **Cd4** > BAPO > **Cd6**~**Cd2** > CARET (Table 2). RT-FTIR monitoring for curves 4, 7, 8 and 9 were stopped before 800 s as very poor kinetics were observed (conversion below 10% after 200 s), characteristics of very poor PIS efficiencies (strongly inhibited by oxygen). Clearly, this is straightforwardly related to the absorption properties of the carbazole derivatives as **Cd7** and **Cd1** are the leaders in the efficiency trend and having higher extinction coefficients at 405 nm contrasted with the others (see Table 1).

#### 2.1.3. Free Radical Photopolymerization

• Photopolymerization of Acrylates

**Figure 4:**
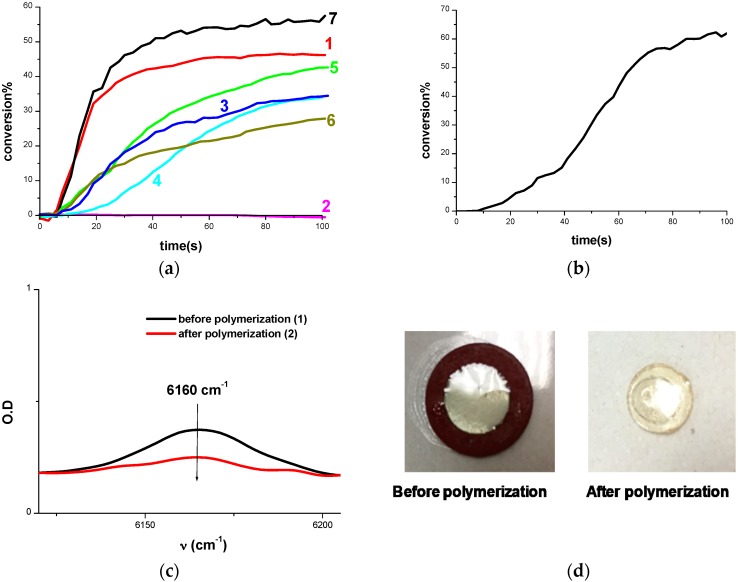
(**a**) Polymerization profiles of TMPTA (acrylate function conversion vs. irradiation time) in laminate upon exposure to LED@405 nm in the presence of: (1) **Cd1**/Iod (0.5%/1% *w*/*w*); (2) **Cd2**/Iod (0.5%/1% *w*/*w*); (3) **Cd3**/Iod (0.5%/1% *w*/*w*); (4) **Cd4**/Iod (0.5%/1% *w*/*w*); (5) **Cd5**/Iod (0.5%/1% *w*/*w*); (6) **Cd6**/Iod (0.5%/1% *w*/*w*); (7) **Cd7**/Iod (0.5%/1% *w*/*w*); (**b**) Polymerization profiles of BisGMA/TEGDMA (70%/30%) (methacrylate function conversion vs. irradiation time) (1.4 mm film thick sample) under air upon exposure to LED@405 nm in the presence of **Cd7**/Iod (0.5%/1% *w*/*w*); (**c**) Near IR spectra recorded before and after polymerization of BisGMA/TEGDMA using **Cd7**/Iod (0.5%/1% *w*/*w*); (**d**) Photos of BisGMA/TEGDMA film (1.4 mm thick) upon irradiation with the LED@405 nm for 100 s in the presence of **Cd7**/Iod (0.5%/1% *w*/*w*) under air before and after polymerization.

The FRP of TMPTA thin films in laminate initiated from diverse Cd/Iod couples is very effective in some cases using the LED@405 nm, while Iod alone obviously does not initiate the polymerization. Typical acrylate function conversion-time profiles are given in Figure 4 and the FCs are outlined in Table 2. Best FCs are achieved when using **Cd7**/Iod and **Cd1**/Iod (~57% and 46% respectively at *t* = 100 s), that’s why these structures will be of more deeply studied in the rest of the manuscript. Strikingly, the reactivity trend follows the order in-line with what observed for the CP process: **Cd7** > **Cd1** > **Cd5** > **Cd3** > **Cd4** > **Cd6**~**Cd2**.

• Photopolymerization of Methacrylates

Interestingly, the **Cd7**/Iod (0.5%/1% *w*/*w*) couple effectively initiates the FRP of a mix of methacrylates (BisGMA/TEGDMA 70%/30% *w*/*w*) under air (1.4 mm thick example) upon illumination with the LED@405 nm, as shown in Figure 4d. Remarkably, a straightforward tack free polymer is produced even in thick examples (Figure 4d).

#### 2.1.4. Synthesis of Inter-Penetrated Networks (IPNs) through Photopolymerization

**Figure 5:**
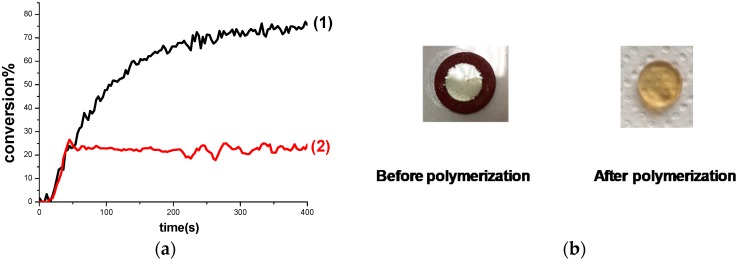
(**a**) Polymerization profiles of TMPTA/EPOX blend (acrylate and epoxy function conversions vs. irradiation time) under air thick sample 1.4 mm upon exposure to LED@405 nm in the presence of **Cd7**/Iod (0.5%/1% *w*/*w*): (1) TMPTA; (2) EPOX; (**b**) Photos of TMPTA/EPOX film (1.4 mm thick) upon irradiation with the LED@405 nm for 400 s in the presence of Cd7/Iod (0.5%/1% *w*/*w*) under air before and after polymerization.

The Cd/Iod two-component PIS can jointly create both free radicals (Ph^•^) and radical cations (Cd^•+^) simultaneously upon visible light exposure using LED@405 nm and hence can be utilized for the synthesis of IPNs from acrylate/epoxy combinations (Figure 5a). Effectively, the synthesis of a thick 1.4 mm tack free coating was performed under air (Figure 5b). Very high final function conversion of acrylate functional group compared to the epoxy functional group (FCs ≈ 76% and 23% for the acrylate and the epoxide, band 1 and band 2 in Figure 5a, respectively). In reality, this behaviour which can be credited to the more receptive radicals (Ph^•^) in the event of free radical polymerization contrasted with cationic polymerization (where cations are starting species) is reported in the literature [19].

#### 2.1.5. Surface Patterning or 3D Printing Using **Cd1**–**Cd7** Based System upon LED@405 nm Projector

**Figure 6:**
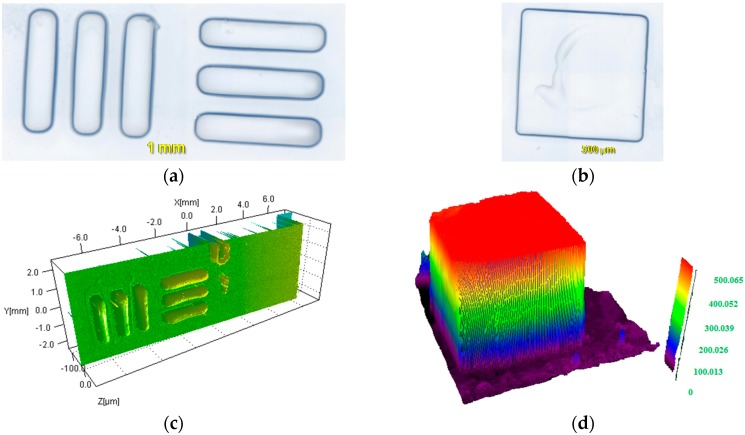
Cationic photopolymerization experiments using LED projector @405 nm: (**a**,**b**) patterns (**c**) Profilometry characterization for a thin pattern obtained (thin samples 0.02 mm); (**d**) observation (numerical optical microscopy) of pattern written in 3D for a thicker sample (0.5 mm).

For 3D printing tests, a LED projector @405 nm (110 mW/cm^2^, Thorlabs, Gmbh, Germany) was utilized. A similar intensity on the surface of the specimen and a similar emission spectrum for the LEDs used in 3D printing and in monitoring FTIR tests were used for purpose of comparison. In Figure 6, some 3D printing tests were done using the LED projector illumination using the **Cd7**/Iod (0.5%/1% *w*/*w*) PIS, which is extremely responsive in the cationic polymerization of epoxides (see above) under air. Remarkably, the high photosensitivity of this resin permits an effective polymerization process in the illuminated area. This LED projector tests demonstrate a favorable element over other laser based 3D printing techniques as the whole layer is illuminated at one time. The fast cationic process upon exposure to the LED projector @405 nm likely outperform the radical process by diminishing the shrinkage generally observed in the latter. Thin 3D objects (25–50 μm) and in addition thicker ones (0.45 mm) can be effectively generated through the LED projector technique (Figure 6).

### 2.2. Photochemical Mechanisms

#### 2.2.1. Steady State Photolysis

**Figure 7:**
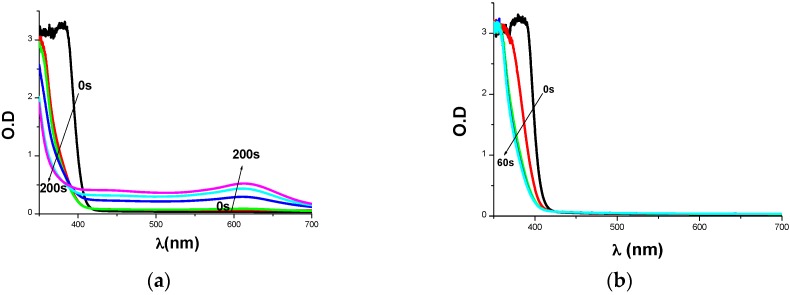
(**a**) **Cd7**/Iod photolysis upon exposure to LED@405 nm and (**b**) Photolysis of **Cd7** in the absence of Iod.

The steady state photolysis of **Cd1**–**Cd7**/Iod in acetonitrile is quick compared to the photolysis of Cds alone (e.g., **Cd7**/Iod in Figure 7a versus **Cd7** alone in Figure 7b). A new photo-product (described by a noteworthy new absorption for λ > 550 nm; Figure 7a) is formed and expected due to the Cd7/Iod.

#### 2.2.2. Fluorescence Quenching, Laser Flash Photolysis (LFP), Cyclic Voltammetry and ESR Experiments

**Figure 8:**
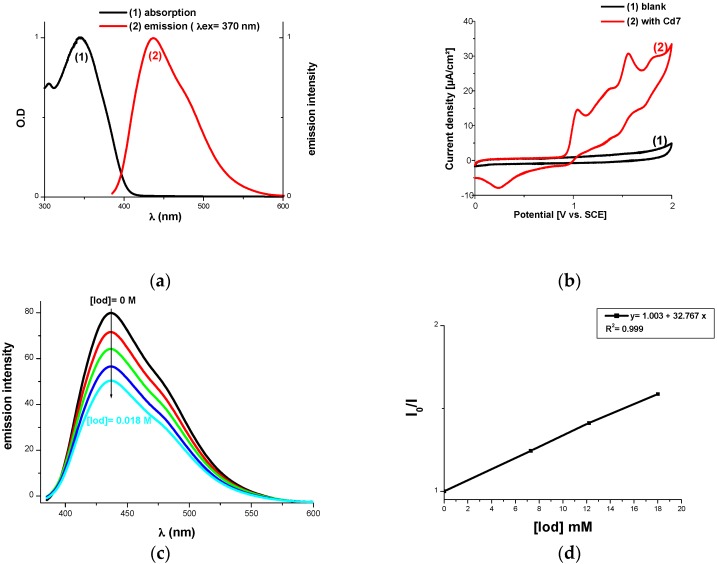
(**a**) Singlet state energy determination; (**b**) Cyclic voltammetry for the Cd7 oxidation; (**c**) Fluorescence quenching of Cd7 with Iod; (**d**) Stern-Volmer treatment of the lifetime quenching of *Cd7 in the presence of Iod.

Fluorescence experiments in acetonitrile are shown in Figure 8 for **Cd7**. The intersection point of the absorption and fluorescence spectra permits the determination of the first singlet excited state energies (E_S1_) (Table 3; e.g., E_S1_ for **Cd7** Figure 8a). Ideal fluorescence quenching processes were proven by high estimations of the Stern Volmer coefficients leading to Φet in (Equation (1)) (Table 3, Figure 8c,d). Highly favorable free energy changes for the electron transfer reaction were noted between carbazole derivatives and Iod (e.g., ΔGet = −1.95 eV for **Cd7**; the oxidation potential for **Cd7** was determined by cyclic voltammetry in Figure 8b), electron transfer quantum yields from S1 (Φet) were computed by Equation (1): Φet ~ 0.43 with **Cd7**). Efficient processes can also be expected from the triplet state of Cd (ΔGet (T1) < 0 eV; Table 3); but the singlet state pathway is probably more favorable due to the high value of Φet from S1 found above:

Φet = Ksv [Iod]/(1 + Ksv[Iod])(1)

The favorable quenching process of carbazole derivatives by Iod is depicted by the reactions r1 and r2:

Cd→ *Cd (hυ)(r1)

*Cd + Ar_2_I^+^ → Cd^●+^ + Ar_2_I^●^ → Cd^●+^ + Ar^●^ + ArI (r2)

**Figure 9:**
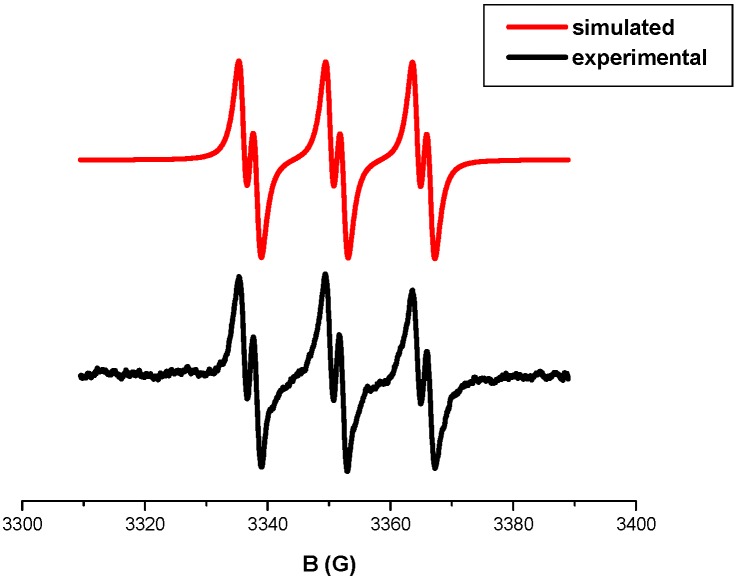
ESR-ST spectra obtained upon irradiation (LED@405 nm) of a **Cd7**/Iod solution, experimental (lower spectrum), and simulated (upper spectrum) in *tert*-butyl benzene as a solvent.

The presence of Ar^●^ (r2) is fully confirmed by ESR results. Indeed, the phenyl radicals (Ar^●^) were easily detected as PBN/Ar^●^ radical adducts in the irradiation of a **Cd7**/Iod solution in ESR-ST experiments (Figure 9) i.e., the simulation of the experimental ESR spectrum yields the hyperfine coupling constants (hfcs): a_N_ = 14.13 G and a_H_ = 2.21 G typical for the PBN/Ph^●^ radical adducts.

#### 2.2.3. Structure/Reactivity/Efficiency Relationship

In both CP and FRP, the studied carbazole derivatives follow the same trend of efficiency in terms of final conversion of both acrylate functional groups as well as epoxy functional groups, respectively. In fact, the efficiency trend of the carbazole derivatives more or less follows their absorption trend. Clearly, both **Cd7** and **Cd1** are the best photoinitiators in terms of efficiency under LED@405 nm irradiation (see above in parts 2 and 3), and at the same time they are characterized by relatively higher absorption ability at this range of wavelengths. On the other hand, the ΔGs of electron transfer of all Cds as well electron transfer quantum yields (Table 3) are relatively similar and thus can be excluded for being the key factor for the Structure/Reactivity/Efficiency relationships of these derivatives as they are rather similar in the **Cd1**–**Cd7** series. Therefore, the key factor affecting the reactivity and efficiency of these carbazole derivatives as PIs is their absorption properties since as better absorption of **Cd7** and **Cd1** of the light from LED@405 nm is observed, these carbazole derivatives exhibit the best performance. The following table (Table 4) summarizes the trends in magnitude of absorption, efficiencies in both CP and FRP.

## 3. Materials and Methods

### 3.1. Chemical Compounds

**Scheme 2:**
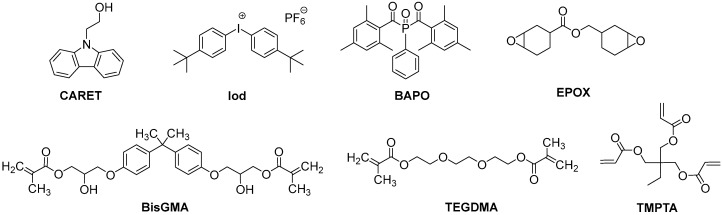
Other used chemical compounds.

9*H*-Carbazole-9-ethanol (CARET) and phenyl-*N*-*tert*-butylnitrone (PBN) (Scheme 2) were obtained from Sigma Aldrich (St. Louis, MO, USA). Bis-(4-tert-butylphenyl)iodonium hexafluorophosphate (Iod or Speedcure 938) and bis(2,4,6-trimethylbenzoyl) phenylphosphine oxide (BAPO or speedcure BPO) were obtained from Lambson (Lambson Ltd., Wetherby, UK). Trimethylolpropane triacrylate (TMPTA) and 3,4-epoxycyclohexylmethyl 3,4-epoxycyclo-hexylcarboxylate (EPOX; Uvacure 1500) were obtained from Allnex (Frankfurt, Germany) and used as benchmark monomers for radical and cationic photopolymerization. Bisphenol A-glycidyl methacrylate (BisGMA) and triethyleneglycol dimethacrylate (TEGDMA) were obtained from Sigma Aldrich and used with the highest purity available. The full procedures for the synthesis of **Cd1**–**Cd7**, are presented in the Supporting Information.

### 3.2. Irradiation Sources

The following Light Emitting Diodes (LEDs) were used as irradiation sources: (i) LED@375 nm—incident light intensity at the sample surface: I_0_ ≈ 40 mW cm^−2^; (ii) LED@405 nm (I_0_ ≈ 110 mW cm^−2^).

### 3.3. Free Radical (FRP) and Cationic (CP) Photopolymerization

The two-component photoinitiating systems (PISs) are mainly based on Cds/Iodonium salt (0.5%/1% *w*/*w*) for both CP and FRP. The weight percent of the photoinitiating system is calculated from the monomer content. The photosensitive thin-film formulations (~25 µm of thickness) were deposited on BaF_2_ pellets under air for the CP of EPOX while for the FRP of TMPTA and BisGMA/TEGDMA, it was done in laminate (the formulation is sandwiched between two polypropylene films to reduce the O_2_ inhibition). The 1.4 mm thick samples of BisGMA/TEGDMA were polymerized under air.

Excellent solubility of all carbazole derivatives except for **Cd6** were observed in EPOX monomer. The decrease of the epoxy group content and the double bond content of (meth)acrylate functions were continuously followed by real time FTIR spectroscopy (JASCO FTIR 4100, Oklahoma City, OK, USA) at about 790 cm^−1^ and 1630 cm^−1^, respectively. The evolution of the methacrylate characteristic peak for the thick samples (1.4 mm) was followed in the near infrared range at ~6160 cm^−1^. The procedure used to monitor the photopolymerization profile has been described in detail in [20,21].

### 3.4. Redox Potentials

The oxidation potentials (Eox vs. SCE) for the different carbazole derivatives were measured in acetonitrile by cyclic voltammetry with tetrabutylammonium hexafluorophosphate 0.1 M as the supporting electrolyte. The free energy change ∆Get for an electron transfer reaction was calculated from the Rehm-Weller equation (Equation (2)) [22] where Eox, Ered, E* and C are the oxidation potential of the electron donor, the reduction potential of the electron acceptor, the excited state energy level and the coulombic term for the initially formed ion pair, respectively. C is neglected as usually done in polar solvents:

∆Get = Eox − Ered − E* + C(2)

### 3.5. ESR Spin Trapping (ESR-ST) Experiments

The ESR-ST experiments were carried out using an X-Band spectrometer (EMX-Plus, Bruker, Billerica, MA, USA). LED@405 nm was used as irradiation source for triggering the production of radicals at room temperature (RT) for N_2_ saturated toluene solutions and trapped by phenyl-*N*-*tert*-butylnitrone (PBN) according to a procedure described by us in details [23]. The ESR spectra simulations were carried out with the PEST WINSIM program.

### 3.6. Absorption Experiments

The UV-vis absorption properties of the compounds were studied using a JASCO V730 spectrophotometer.

### 3.7. Fluorescence Experiments

The fluorescence properties of the compounds were studied using a JASCO FP-6200 fluorimeter. 

### 3.8. Computational Procedure

Molecular orbital calculations were carried out with the Gaussian 03 suite of programs [24,25]. The electronic absorption spectra for the different compounds were calculated with the time-dependent density functional theory at the MPW1PW91/6-31g(d) level of theory on the relaxed geometries calculated at the UB3LYP/6-31G* level of theory.

### 3.9. 3D Printing Experiments

For 3D printing experiments, a LED projector @405 nm (Thorlabs) was used. The photosensitive cationic resin was polymerized under air and the generated patterns analyzed by an opto-digital microscope (DSX-HRSU from Olympus Corporation, Tokyo, Japan) or by profilometry. The procedure was presented in [26].

## 4. Conclusions

In the present paper, the carbazole scaffold is proposed as an excellent precursor for the development of new photoinitiators using near-UV and visible wavelength LEDs for the photoinitiation of both the cationic polymerization of epoxides and the free radical polymerization of (meth)acrylates. High final conversions and polymerization rates are achieved. Examples of using these new initiating frameworks in cationic photocurable resins for LED projector 3D printing are given. Basically, the primary processes involve the simultaneous production of radicals and radical cations that can be applied both in radical chemistry and/or cationic chemistry. New photoinitiating systems based on carbazole derivatives capable to be operated at various illumination wavelengths or that can be incorporated in 3D printing resins will be reported in future papers.

## Figures and Tables

**Table 1:** Absorption properties for **Cd1**–**Cd7** vs. CARET and BAPO (@405 nm).

	Cd1	Cd2	Cd3	Cd4	Cd5	Cd6	Cd7	BAPO	CARET
ε ^1^	1000	n.a ^2^	180	90	140	n.a ^2^	1500	500	n.a ^2^

^1^ @405 nm (M^−1^·cm^−1^); ^2^ no absorption.

**Table 2:** Function conversions (FC): epoxy for EPOX; acrylate for TMPTA and methacrylate for BisGMA/TEGDMA using different photoinitiating systems; LED@405 nm for irradiation.

	Cd1	Cd2	Cd3	Cd4	Cd5	Cd6	Cd7	BAPO	CARET
EPOX ^1^	44%	0%	41%	27%	30%	37%	44%	10%	1%
TMPTA ^2^	46%	0%	34%	34%	43%	28%	57%		60%
BisGMA/TEGDMA ^3^	57%	0%					60%		

^1^ Epoxy function Conversion FC (%) for EPOX (at *t* = 100 s) (0.5% PI + 1% Iod); ^2^ Acrylate function conversion FC for TMPTA (%) (at *t* = 100 s) (0.5% PI + 1% Iod); excepted (1% CARET + 1% Iod); ^3^ Methacrylate conversion FC for BisGMA/TEGDMA (%) (at *t* = 100 s) (0.5% PI + 1% Iod).

**Table 3:** Parameters characterizing the chemical mechanisms associated with the **Cd1**–**Cd7**/Iod systems in acetonitrile.

	Eox (eV) ^1^	E_S1_ (eV)	ΔG_etS1_ (eV)	E_T1_ (eV) ^2^	ΔG_etT1_^2^ (eV)	Φet (S1)
**Cd1**	0.92	3.17	−2.05	1.97	−0.85	0.34
**Cd2**	1.06	3.5	−2.22	1.99	−0.73	0.5
**Cd3**	0.97	3.2	−2.02	1.97	−0.81	0.41
**Cd4**	0.96	3.35	−2.19	1.99	−0.83	0.5
**Cd5**	0.99	3.25	−2.06	1.97	−0.79	
**Cd6**	1.49	3.5	−1.81	3.04	−1.36	
**Cd7**	0.98	3.13	−1.95	1.89	−0.72	0.43

^1^ Oxidation potentials for **Cd1**–**Cd7** (this work); ^2^ from molecular orbital calculations (uB3LYP/6-31G* level of theory).

**Table 4:** Trends of bathochromic shift, Cationic polymerization efficiency, and Free radical polymerization efficiency.

**Trend of bathochromic shift**	**Cd7**~**Cd1** > **Cd3** > **Cd5** > **Cd4** > **Cd2** > **Cd6**
**Trend of CP efficiency**	**Cd7**~**Cd1** > **Cd3** > **Cd5** > **Cd4** > **Cd6**~**Cd2**
**Trend of FRP efficiency**	**Cd7** > **Cd1** > **Cd5** > **Cd3** > **Cd4** > **Cd6**~**Cd2**

## Supplementary Materials

Supplementary Materials are available online, the synthesis of **Cd1**–**Cd7**, the procedures are fully presented in supporting information.
