# Supplementary material for: Novel Carbazole Skeleton-Based Photoinitiators for LED Polymerization and LED Projector 3D Printing
Source: Molecules. 2017 Dec 4;22(12):2143. doi: 10.3390/molecules22122143 (PMC6149745; doi:10.3390/molecules22122143)
Supplement: Supplementary file 1 [file molecules-22-02143-s001.pdf]

molecules

MDPI

Novel carbazole Scaffold Based Photoinitiators for LED polymerization and LED Projector 3D Printing.

Assi Al Mousawi[1,2], Patxi Garra[1], Frederic Dumur*[3], Thanh-Tuan Bui[4], Fabrice Goubard[4], Joumana Toufaily[2], Tayssir Hamieh[2], Bernadette Graff[1], Didier Gigmes[3], Jean Pierre Fouassier[1], Jacques Lalevée*[1]

[1] Institut de Science des Matériaux de Mulhouse IS2M – UMR CNRS 7361 – UHA, 15, rue Jean Starcky, 68057 Mulhouse Cedex, France.

[2] Laboratoire de Matériaux, Catalyse, Environnement et Méthodes analytiques (MCEMA-CHAMSI), EDST, Université Libanaise, Campus Hariri, Hadath, Beyrouth, Liban.

[3] Aix Marseille Univ, CNRS, ICR UMR 7273, F-13397 Marseille, France.

[4] Laboratoire de Physicochimie des Polymères et des Interfaces LPPI, Université de Cergy-Pontoise, 5 mail Gay Lussac, Neuville-sur-Oise, 95031 Cergy-Pontoise Cedex, France.

* Correspondence: jacques.lalevee@uha.fr; frederic.dumur@univ-amu.fr

Supporting Information

All reagents and solvents were purchased from Aldrich or Alfa Aesar and used as received without further purification. Mass spectroscopy was performed by the Spectropole of Aix-Marseille University. ESI mass spectral analyses were recorded with a 3200 QTRAP (Applied Biosystems SCIEX) mass spectrometer. The HRMS mass spectral analysis was performed with a QStar Elite (Applied Biosystems SCIEX) mass spectrometer. Elemental analyses were recorded with a Thermo Finnigan EA 1112 elemental analysis apparatus driven by the Eager 300 software. ^{1}H and ^{13}C NMR spectra were determined at room temperature in 5 mm o.d. tubes on a Bruker Avance 400 spectrometer of the Spectropole: ^{1}H (400 MHz) and ^{13}C (100 MHz). The ^{1}H chemical shifts were referenced to the solvent peaks $CDCl_3$ (7.26 ppm), DMSO (2.49 ppm) and the ^{13}C chemical shifts were referenced to the solvent peak $CDCl_3$ (77 ppm), DMSO (49.5 ppm). All these carbazole photoinitiators were prepared with analytical purity up to accepted standards for new organic compounds (>98%) which was checked by high field NMR analysis.

Figure S1. Synthetic pathways to **Cd1-Cd7**.

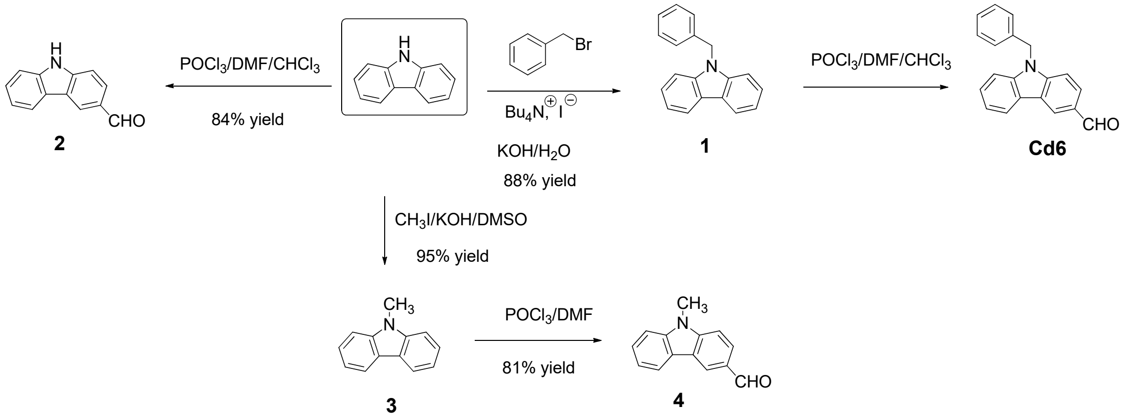
POCl3/DMF/CHCl3
84% yield
CHO
2
Br
Bu4N I
KOH/H2O
88% yield
1
POCl3/DMF/CHCl3
CHO
Cd6
CH3I/KOH/DMSO
95% yield
CH3
3
POCl3/DMF
81% yield
CHO
4

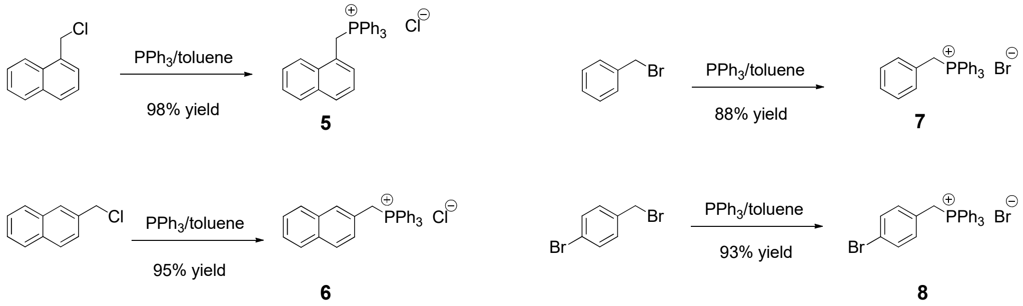
Cl
PPh3/toluene
98% yield
PPh3 Cl
5
Br
PPh3/toluene
88% yield
PPh3 Br
7
Cl
PPh3/toluene
95% yield
PPh3 Cl
6
Br
Br
PPh3/toluene
93% yield
Br
PPh3 Br
8

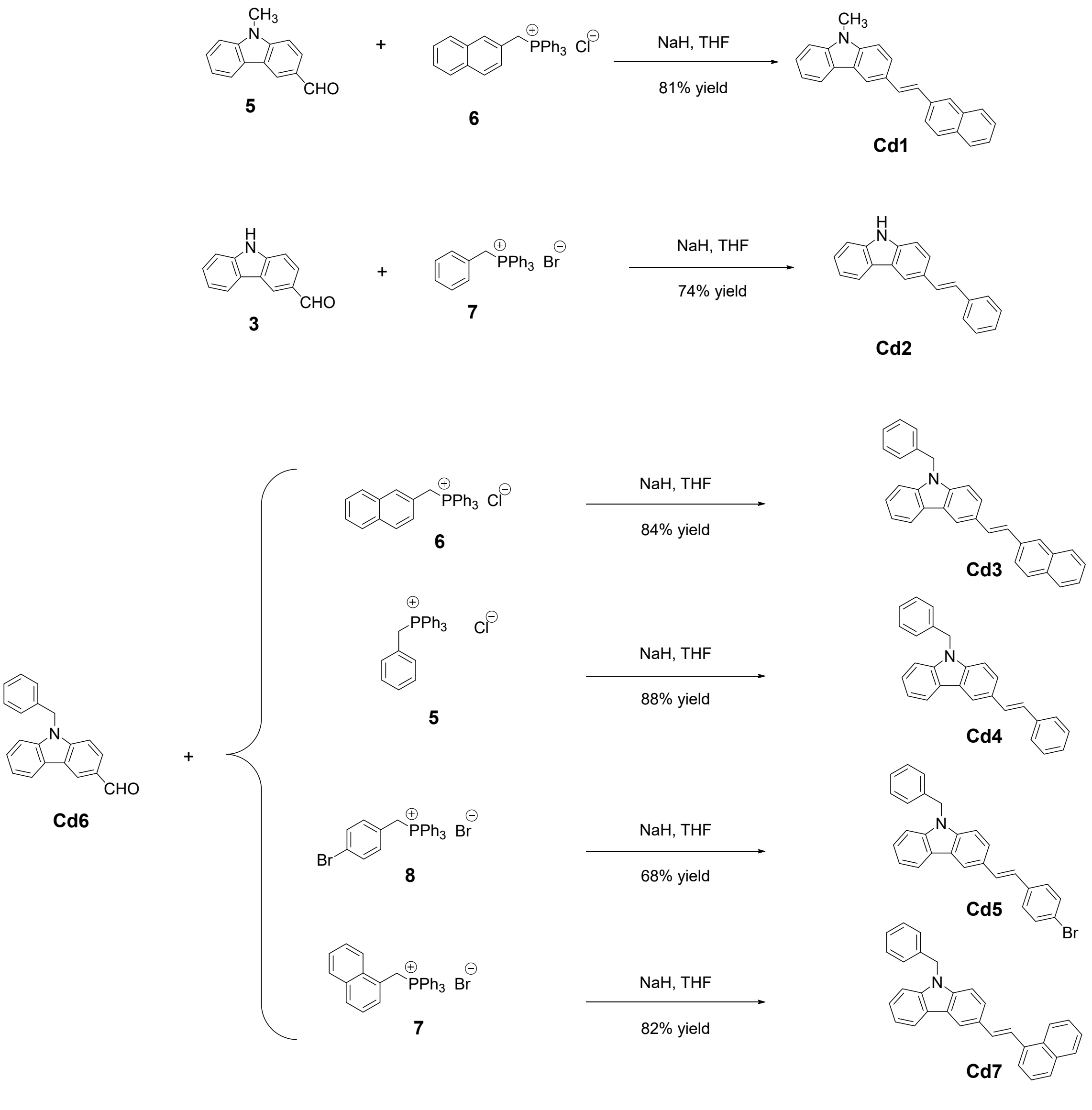

Synthesis of (E)-9-methyl-3-(2-(naphthalen-2-yl)vinyl)-9H-carbazole ***Cd1***

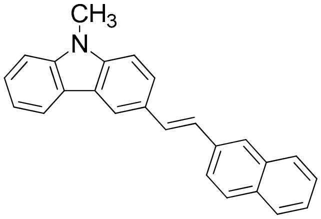

To a suspension of 9-methyl-9*H*-carbazole-3-carbaldehyde (0.48 g, 2.28 mmol) and (naphthalen-2-ylmethyl)triphenylphosphonium chloride **6** (1 g, 2.28 mmol) in 100 mL dry THF was added sodium hydride (1 g, 41.67 mmol). The solution was refluxed overnight. During reflux, the color changed from white to light yellow and orange. The residue was quenched with water. The solution was extracted several times with DCM, the organic phases were combined, dried over magnesium sulfate and the solvent removed under reduced pressure. Addition of ethanol precipitated a white solid that was filtered off, washed with cold ethanol and dried under vacuum

(615 mg, 81% yield). ^{1}H NMR ($CDCl_3$) δ 3.90 (s, 3H), 7.28-7.32 (m, 1H), 7.34 (d, 1H, J = 16.2 Hz), 7.42-7.55 (m, 7H), 7.76-7.86 (m, 6H), 7.90 (d, 1J, J = 16.2 Hz), 8.17 (d, 1H, J = 7.7 Hz), 8.31 (d, 1H, J = 1.6 Hz); ^{13}C NMR ($CDCl_3$) δ 29.2, 108.66, 108.71, 118.7, 119.2, 120.4, 122.9, 123.2, 123.6, 124.6, 125.6, 125.9, 126.0, 126.1, 126.3, 127.7, 127.9, 128.3, 128.6, 130.1, 132.8, 133.9, 135.5, 140.9, 141.5; HRMS (ESI MS) m/z: theor: 333.1517 found: 333.1513 ($[M]^{+\cdot}$ detected)

Synthesis of (E)-9-methyl-3-styryl-9H-carbazole ***Cd2***

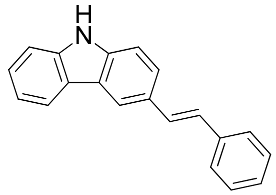

To a suspension 9*H*-carbazole-3-carbaldehyde (445 mg, 2.28 mmol) and benzyltriphenylphosphonium bromide **7** (987 mg, 2.28 mmol) in 100 mL dry THF was added sodium hydride (1 g, 41.67 mmol). The solution was refluxed overnight. During reflux, the color changed from white to light yellow. The residue was quenched with water. The organic phases were combined, dried over magnesium sulfate and the solvent removed under reduced pressure. The residue was purified by column chromatography (SiO_2) using DCM:pentane 1:1 as the eluent (454 mg, 74% yield). ^{1}H NMR ($CDCl_3$) δ 1.75 (brs, 1H, NH), 7.09 (d, 1H, J = 14.5 Hz), 7.31-7.56 (m, 9H), 7.70-7.76 (m, 2H), 8.12 (d, 2H, J = 7.5 Hz); ^{13}C NMR ($CDCl_3$) δ 110.6, 119.4, 119.8, 120.30, 120.33, 120.7, 123.3, 124.1, 125.79, 125.83, 126.3, 127.2, 128.9, 129.0, 129.6, 136.3, 139.5; HRMS (ESI MS) m/z: theor: 269.1204 found: 269.1205 ($[M]^{+\cdot}$ detected).

Synthesis of (E)-9-benzyl-3-(2-(naphthalen-2-yl)vinyl)-9H-carbazole ***Cd3***

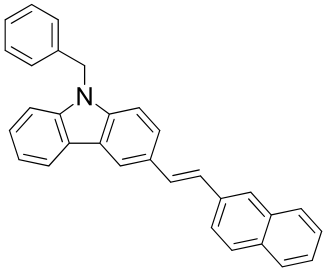

To a suspension of 9-benzyl-9*H*-carbazole-3-carbaldehyde (0.65 g, 2.28 mmol) and (naphthalen-2-ylmethyl)triphenylphosphonium chloride **6** (1 g, 2.28 mmol) in 100 mL dry THF was added sodium hydride (1 g, 41.67 mmol). The solution was refluxed overnight. During reflux, the color changed from white to light yellow and orange. The residue was quenched with water. The solution was extracted several times with DCM, the organic phases were combined, dried over magnesium sulfate and the solvent removed under reduced pressure. Addition of ethanol precipitated a white solid that was filtered off, washed with cold ethanol and dried under vacuum (784 mg, 84% yield). ^{1}H NMR (DMSO-d_6) δ 5.53 (s, 2H), 7.16 (d, 2H, J = 7.9 Hz), 7.26-7.30 (m, 4H), 7.34-7.38 (m, 3H), 7.43-7.47 (m, 4H), 7.69 (dd, 1H, J = 8.5 Hz, J = 1.4 Hz), 7.79-7.89 (m, 5H), 8.18 (d, 1H, J = 7.7 Hz), 8.32 (s, 1H); ^{13}C NMR (DMSO-d_6) δ 46.7, 109.14, 109.20, 118.7, 119.5, 120.5, 123.1, 123.5, 123.6, 124.7, 125.6,

126.0, 126.1, 126.3, 126.37, 126.42, 127.5, 127.7, 127.9, 128.3, 128.8, 129.1, 129.9, 132.8, 133.9, 135.5, 137.0, 140.5, 141.1; HRMS (ESI MS) m/z: theor: 409.1830 found: 409.1831 ($[M]^{+\cdot}$ detected).

Synthesis of (E)-9-benzyl-3-styryl-9H-carbazole ***Cd4***

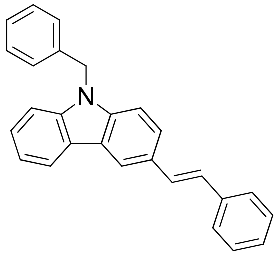

To a suspension of 9-benzyl-9*H*-carbazole-3-carbaldehyde (650 mg, 2.28 mmol) and benzyltriphenylphosphonium bromide **5** (987 mg, 2.28 mmol) in 100 mL dry THF was added sodium hydride (1 g, 41.67 mmol). The solution was refluxed overnight. During reflux, the color changed from white to light yellow. The residue was quenched with water. The organic phases were combined, dried over magnesium sulfate and the solvent removed under reduced pressure. The residue was purified by column chromatography (SiO_2) using DCM:pentane 1:1 as the eluent (720 mg, 88% yield). ^1H NMR (DMSO-d_6) δ 2.49 (s, 2H), 7.18-7.29 (m, 8H), 7.34-7.45 (m, 4H), 7.60-7.73 (m, 5H), 8.20 (d, 1H, J = 7.4 Hz), 8.42 (s,1H); ^{13}C NMR (DMSO-d_6) δ 45.7, 109.80, 109.83, 118.6, 119.3, 120.5, 122.3, 122.7, 124.9, 125.8, 126.0, 126.12, 126.7, 127.1, 127.3, 128.5, 128.6, 128.7, 129.3, 137.6, 137.7, 140.0, 140.6; HRMS (ESI MS) m/z: theor: 359.1674 found: 359.1678 ($[M]^{+\cdot}$ detected).

Synthesis of (E)-9-benzyl-3-(4-bromostyryl)-9H-carbazole ***Cd5***

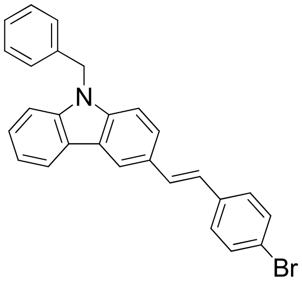

To a suspension of 9-benzyl-9*H*-carbazole-3-carbaldehyde (650 mg, 2.28 mmol) and 4-bromobenzyltriphenylphosphonium bromide **8** (1.17 g, 2.28 mmol) in 100 mL dry THF was added sodium hydride (1 g, 41.67 mmol). The solution was refluxed overnight. During reflux, the color changed from white to light yellow. The residue was quenched with water. The organic phases were combined, dried over magnesium sulfate and the solvent removed under reduced pressure. The residue was purified by column chromatography (SiO_2) using DCM:pentane 1:1 as the eluent (678 mg, 68% yield). ^1H NMR (DMSO-d_6) δ 5.66 (s, 2H), 7.18-7.26 (m, 7H), 7.42-7.73 (m, 9H), 8.18 (d, 1H, J = 7.4 Hz), 8.42 (s, 1H); ^{13}C NMR (DMSO-d_6) δ 45.7, 109.81, 109.85, 118.7, 119.3, 119.7, 120.4, 122.3, 122.6, 124.5, 124.9, 126.1, 126.7, 127.3, 128.0, 128.3, 128.6, 130.3, 131.6, 137.0, 137.7, 140.1, 140.5; HRMS (ESI MS) m/z: theor: 437.0779 found: 437.0781 ($[M]^{+\cdot}$ detected).

Synthesis of 9-benzyl-9H-carbazole-3-carbaldehyde ***Cd6***

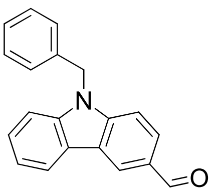

A solution of $CHCl_3$ (25 mL) containing 9-benzyl-9*H*-carbazole (6.43 g, 25 mmol) and DMF (1.86 mL) was cooled to 0°C. $POC1_3$ (2.3 mL) was slowly added at 0°C and the solution was allowed to stir to room temperature for one hour. Then, the solution mixture was refluxed for 16 h. During reaction, a yellow precipitate formed. After cooling, the solution was poured into ice water. The yellow solid was filtered off, washed with ether and pentane, and dried under vacuum. The crude product was recrystallized in ethanol, and cooled at -30°C to end the precipitation (5.41 g, 76% yield). ^{1}H NMR (DMSO-d_6) δ 5.75 (s, 2H), 7.18-7.35 (m, 6H), 7.52 (t, 1H, J = 7.4 Hz), 7.72 (d, 1H, J = 8.2 Hz), 7.83 (d, 1H, J = 8.6 Hz), 7.99 (d, 1H, J = 8.5 Hz), 8.32 (d, 1H, J = 7.8 Hz), 8.79 (s, 1H), 10.1 (s, 1H, CHO); ^{13}C NMR (DMSO-d_6) δ 45.8, 110.0, 110.3, 120.4, 120.8, 122.4, 122.4, 123.9, 126.7, 126.80, 126.84, 127.4, 128.57, 128.61, 137.1, 140.9, 143.7, 191.8; ^{1}H NMR ($CDCl_3$) δ 5.57 (s, 2H), 7.13-7.15 (m, 2H), 7.26-7.29 (m, 2H), 7.35 (td, 1H, J = 7.9 Hz, J = 0.9 Hz), 7.41-7.52 (m, 4H), 7.97-7.99 (m, 1H), 8.20 (d, 1H, J = 7.7 Hz), 8.66 (d, 1H, J = 1.2 Hz), 10.1 (s, 1H, CHO); HRMS (ESI MS) m/z: theor: 285.1154 found: 285.1155 ($[M]^{+\cdot}$ detected).

Synthesis of (E)-9-benzyl-3-(2-(naphthalen-1-yl)vinyl)-9H-carbazole ***Cd7***

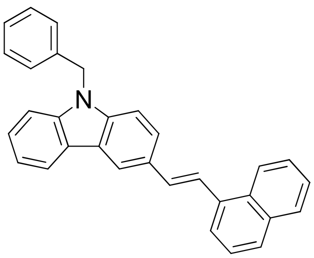

To a suspension of 9-benzyl-9*H*-carbazole-3-carbaldehyde (0.65 g, 2.28 mmol) and (naphthalen-1-ylmethyl)triphenylphosphonium chloride **7** (1 g, 2.28 mmol) in 100 mL dry THF was added sodium hydride (1 g, 41.67 mmol). The solution was refluxed overnight. During reflux, the color changed from white to light yellow. The residue was quenched with water. The organic phases were combined, dried over magnesium sulfate and the solvent removed under reduced pressure. The residue was purified by column chromatography (SiO_2) using DCM:pentane 1:1 as the eluent (765 mg, 82% yield). ^{1}H NMR (DMSO-d_6) δ 5.69 (s, 2H), 7.19-7.29 (m, 6H), 7.43-7.68 (m, 7H), 7.86-7.97 (m, 4H), 8.11 (d, 1H, J = 16.0 Hz), 8.28 (d, 1H, J = 7.7 Hz), 8.49 (d, 1H, J = 8.0 Hz), 8.62 (s, 1H); ^{13}C NMR (DMSO-d_6) δ 45.7, 109.8, 118.9, 119.3, 120.5, 122.2, 122.4, 122.6, 122.7, 123.9, 125.4, 125.9, 126.1, 126.7, 127.27, 127.31, 128.4, 128.5, 128.6, 128.8, 130.8, 132.2, 133.5, 134.8, 137.7, 140.1, 140.6; HRMS (ESI MS) m/z: theor: 409.1830 found: 409.1833 ($[M]^{+\cdot}$ detected).

Synthesis of 9-benzyl-9H-carbazole **1**

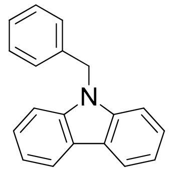

Into a 100 mL one-necked round bottom flask were added carbazole (4.3 g, 17.9 mmol), benzyl bromide (2.56 g, 15.0 mmol), tetrabutylammonium iodide (0.55 g, 0.15 mmol), 50 wt% of aqueous KOH solution (30 mL) and toluene (30 mL). The reaction mixture was heated to reflux overnight. After cooling to room temperature, the reaction mixture was extracted with toluene. The organic layer was washed with water and dried over magnesium sulfate. A white solid was obtained in 88% yield (3.39 g) and used without any further purification. ^{1}H NMR ($CDCl_3$) δ 8.13 (d, 2H, J = 7.7 Hz), 7.45–7.37 (m, 4H), 7.35–7.23 (m, 5H), 7.14 (d, 2H, J = 7.6 Hz), 5.51 (s, 2H); ^{13}C NMR ($CDCl_3$) δ 46.7, 109.0, 119.4, 120.5, 123.2, 126.0, 126.6, 127.6, 128.9, 137.4, 140.9; HRMS (ESI MS) m/z: theor: 257.1204 found: 257.1203 ($[M]^{+\cdot}$ detected).

Synthesis of 9H-carbazole-3-carbaldehyde **2**

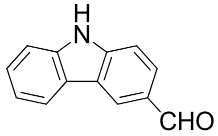

A solution of $CHCl_3$ (25 mL) containing 9*H*-carbazole (4.18 g, 25 mmol) and DMF (1.86 mL) was cooled to 0°C. $POC1_3$ (2.3 mL) was slowly added at 0°C and the solution was allowed to stir to room temperature for one hour. Then, the solution mixture was refluxed for 16 h. After cooling, the solution was poured into ice water. The white solid was filtered off, washed with ether and pentane, and dried under vacuum. The crude product was recrystallized by dissolution in DCM and precipitation in pentane (4.09 g, 84% yield). ^{1}H NMR ($CDCl_3$) δ 7.25-7.34 (m, 1H); 7.43-7.54 (m, 1H), 7.61-7.65 (m, 2H), 7.93-8.05 (m, 1H), 8.26 (d, 1H, J = 7.8 Hz), 8.67-8.74 (m, 1H), 10.10 (s, 1H, CHO), 10.90 (brs, 1H, NH); ^{13}C NMR ($CDCl_3$) δ 112.3, 112.5, 121.1, 121.5, 124.0, 124.2, 124.9, 127.3, 127.6, 130.1, 141.7, 144.7, 192.1; HRMS (ESI MS) m/z: theor: 195.0684 found: 195.0689 ($[M]^{+}$ detected).

Synthesis of 9-methyl-9H-carbazole **3**

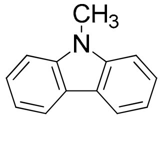

1-Iodomethane (14.2 g, 100 mmol) and potassium hydroxide (33.9 g, 605.4 mmol) were added to a solution of carbazole (18.5 g, 101.1 mmol) in DMSO (200 mL) under stirring. The reaction mixture

was stirred for 24 h at 80°C. The reaction was quenched with water. A white precipitate formed. It was filtered off, washed several times with water and pentane, and finally dried under vacuum. It was purified by filtration on a plug of silicagel using DCM as the eluent (17.37 g, 95% yield). ^{1}H NMR (DMSO-d$_6$) δ 3.87 (s, 3H), 7.21 (t, 2H, J = 7.2 Hz), 7.47 (t, 2H, J = 7.2 Hz), 7.58 (d, 2H, J = 8.0 Hz), 8.15 (d, 2H, J = 7.5 Hz); ^{13}C NMR (DMSO-d$_6$) δ 28.9, 129.0, 118.6, 120.1, 121.9, 125.6, 140.6; HRMS (ESI MS) m/z: theor: 181.0891 found: 181.0892 ([M]$^+$ detected).

Synthesis of 9-methyl-9H-carbazole-3-carbaldehyde **4**

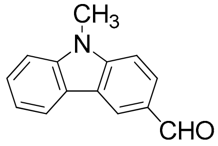

A solution of DMF (25 mL) containing 9-methyl-9*H*-carbazole **3** (4.5 g, 25 mmol) was cooled to 0°C. POC1$_3$ (2.3 mL) was slowly added at 0°C and the solution was allowed to stir to room temperature for one hour. Then, the solution mixture was heated at 130°C for 4 hours. After cooling, the solution was poured into ice water/aq. NaOH solution. Solvents were removed under reduced pressure. Water was added and the solution was extracted with chloroform several times. The organic phases were combined, dried over magnesium sulfate and the solvent removed under vacuum. The residue was purified by column chromatography (SiO_2) using chloroform as the eluent (4.23 g, 81% yield). ^{1}H NMR ($CDCl_3$) δ 3.83 (s, 3H), 7.33 (td, 1H, J = 7.9 Hz, J = 0.9 Hz), 7.39-7.42 (m, 2H), 7.54 (td, 1H, J = 7.1 Hz, J = 1.2 Hz), 7.99 (dd, 1H, J = 8.5 Hz, J = 1.6 Hz), 8.12 (d, 1H, J = 7.7 Hz), 8.55 (d, 1H, J = 1.2 Hz); ^{13}C NMR ($CDCl_3$) δ 29.3, 108.7, 109.1, 110.6, 120.4, 120.6, 122.9, 123.8, 126.8, 127.2, 128.5, 140.7, 144.5, 191.8; HRMS (ESI MS) m/z: theor: 209.0841 found: 209.0844 ([M]$^+$ detected).

Synthesis of (naphthalen-1-ylmethyl)triphenylphosphonium chloride **5**

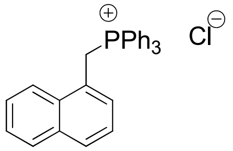

1-Chloromethylnaphthalene (5 g, 28.30 mmol) and triphenylphosphine (7.42 g, 28.30 mmol) were suspended in 50 mL toluene and the solution was refluxed overnight. During reaction, a white precipitate formed. It was filtered off, washed several times with ether and dried under vacuum (12.15 g, 98% yield). ^{1}H NMR (DMSO-d$_6$) δ 5.63 (d, 2H, J = 15.3 Hz), 7.11 (t, 1H, J = 7.7 Hz), 7.29-7.42 (m, 3H), 7.61-7.91 (m, 18H); ^{13}C NMR (DMSO-d$_6$) δ 25.2 (d, J = 47.2 Hz), 117.6 (d, J = 85.1 Hz), 123.8, 125.1 (d, J = 9.2 Hz), 125.9, 128.4, 129.1 (d, J = 9.1 Hz), 129.7 (d, J = 6.8 Hz), 129.9 (d, J = 12.4 Hz), 132.0 (d, J = 4.5 Hz), 133.3 (d, J = 2.7 Hz), 134.0 (d, J = 9.9 Hz), 135.0 (d, J = 2.6 Hz); ^{31}P NMR (DMSO-d$_6$) δ 22.9; HRMS (ESI MS) m/z: theor: 403.1610 found: 403.1607 ([M]$^+$ detected).

Synthesis of (naphthalen-2-ylmethyl)triphenylphosphonium chloride **6**

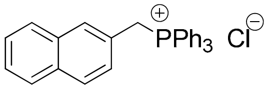

2-Chloromethylnaphthalene (5 g, 28.30 mmol) and triphenylphosphine (7.42 g, 28.30 mmol) were suspended in 250 mL toluene and the solution was refluxed for 3 days. During reaction, a white precipitate formed. It was filtered off, washed several times with ether and dried under vacuum (11.78 g, 95% yield). ^{1}H NMR (DMSO-d_6) δ 5.48 (d, 2H, J = 15.8 Hz), 7.11 (d, 1H, J = 8.5 Hz), 7.48-7.53 (m, 3H), 7.59-7.61 (m, 1H), 7.73-7.79 (m, 13H), 7.86-7.93 (m, 4H); ^{13}C NMR (DMSO-d_6) δ 28.3 (d, J = 46.4 Hz), 117.5, 118.3, 125.5 (d, J = 8.9 Hz), 126.7 (d, J = 9.5 Hz), 127.3, 127.6, 128.1 (d, J = 4.3 Hz), 128.2 (d, J = 2.4 Hz), 130.0, 130.1, 130.3 (d, J = 7.3 Hz), 132.1 (d, J = 5.7 Hz), 132.4 (d, J = 3.3 Hz), 134.0, 134.1, 135.0 (d, J = 2.8 Hz); ^{31}P NMR (DMSO-d_6) δ 23.0; HRMS (ESI MS) m/z: theor: 403.1610 found: 403.1609 ($[M]^+$ detected).

Synthesis of benzyltriphenylphosphonium bromide ***7***

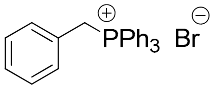

Benzyl bromide (4.84 g, 28.30 mmol) and triphenylphosphine (7.42 g, 28.30 mmol) were suspended in 50 mL toluene and the solution was refluxed overnight. During reaction, a white precipitate formed. It was filtered off, washed several times with ether and dried under vacuum (10.76 g, 88% yield). ^{1}H NMR (DMSO-d_6) δ 5.12 (d, 2H, J = 15.7 Hz), 6.98 (d, 2H, J = 4.9 Hz), 7.25 (d, 3H, J = 7.3 Hz), 7.91-7.63 (m,15H); HRMS (ESI MS) m/z: theor: 353.1454 found: 353.1455 ($[M]^+$ detected).

Synthesis of 4-bromobenzyltriphenylphosphonium bromide ***8***

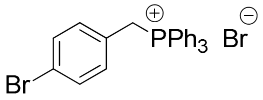

4-Bromobenzyl bromide (7.07 g, 28.30 mmol) and triphenylphosphine (7.42 g, 28.30 mmol) were suspended in 50 mL toluene and the solution was refluxed overnight. During reaction, a white precipitate formed. It was filtered off, washed several times with ether and dried under vacuum (13.42 g, 93% yield). ^{1}H NMR (DMSO-d_6) δ 5.22 (d, 2H, J = 15.8 Hz), 6.91 (d, 2H, J = 6.7 Hz), 7.44 (d, 2H, J = 6.7 Hz), 7.65-7.73 (m, 12H), 7.88-7.90 (m, 3H); HRMS (ESI MS) m/z: theor: 431.0559 found: 431.0555 ($[M]^+$ detected).
